# Silencing METTL14 alleviates liver injury in non-alcoholic fatty liver disease by regulating mitochondrial homeostasis

**DOI:** 10.17305/bb.2023.9698

**Published:** 2024-06-01

**Authors:** Wei Wang, Jun Yan, Long Han, Zi-Lin Zou, Ai-Lei Xu

**Affiliations:** 1 Gastroenterology Department, Hunan Aerospace Hospital, Changsha, China

**Keywords:** Non-alcoholic fatty liver disease (NAFLD), methyltransferase-like 14 (METTL14), mitochondrial homeostasis, imbalance, N6-methyladenosine (m6A) modification, miR-34a-5p

## Abstract

Mitochondrial dysfunction is an important pathogenic factor in non-alcoholic fatty liver disease (NAFLD). Methyltransferase-like 14 (METTL14) has been implicated in mitochondrial fission processes. This research aimed to investigate the mechanism of METTL14 in the mitochondrial function of NAFLD. We first established NAFLD mouse models and cell models, recording body and liver weights and examining pathological changes in liver tissues. Subsequently, serum levels of liver function indices (aspartate aminotransferase [AST], alanine aminotransferase [ALT], total cholesterol [TC], and triglycerides [TG]), inflammatory markers (tumor necrosis factor-alpha [TNF-α], interleukin [IL]-6, and IL-1β), and mitochondrial dysfunction indicators (fission 1 protein [Fis1], dynamin-related protein 1 [Drp1], mitofusin 2 [Mfn2], SID1 transmembrane family member 2 [SIDT2], and mitochondrial membrane potential [MMP]) in the liver and cells were evaluated. The N6-methyladenosine (m6A) modification level of primary microRNA (pri-miRNA) and m6A enrichment on pri-miR-34a were quantified. Co-immunoprecipitation and dual-luciferase reporter gene assays were utilized to validate gene interactions. Our findings revealed highly elevated METTL14 expression in NAFLD mouse and cell models. Silencing METTL14 reduced weight gain and mitigated adverse liver function indices, inflammation, hepatic steatosis, and structural damage in NAFLD mice. It also led to a decrease in Fis1/Drp1 levels and an increase in MMP/Mfn2 in the liver and cells. Moreover, METTL14 increased the m6A level, promoting the binding of DiGeorge syndrome critical region 8 (DGCR8) to pri-miR-34a, which enhanced miR-34a-5p expression. Databases and dual-luciferase reporter gene assays indicated that miR-34a-5p could suppress SIDT2 expression. The overexpression of miR-34a-5p or inhibition of SIDT2 expression negated the alleviative effects of METTL14 silencing on mitochondrial homeostasis imbalance. In conclusion, METTL14, through m6A modification, modulates the miR-34a-5p/SIDT2 axis, impairing mitochondrial homeostasis in NAFLD.

## Introduction

Non-alcoholic fatty liver disease (NAFLD) is a prevalent chronic liver disease with a global incidence of 32.4%. This condition can cause liver fibrosis, cirrhosis, and non-alcoholic steatohepatitis (NASH) [1]. The primary manifestations of NAFLD are liver fat accumulation and hepatic steatosis [2]. Insulin resistance leads to the occurrence of metabolic syndrome, which is a potential risk factor for NAFLD [3]. Currently, pharmacological treatments like vitamin E and pioglitazone are commonly prescribed for NAFLD. However, these treatments have limitations. For instance, vitamin E is not sufficiently effective against fibrosis, while pioglitazone can induce side effects, such as weight gain and bone loss. Additionally, both treatments can increase the risks of bleeding and tumorigenesis [4]. An imbalance in mitochondrial homeostasis can lead to changes in mitochondrial number, morphology, and function, which may subsequently result in the occurrence of various diseases [5]. More importantly, mitochondrial dysfunction is known to exacerbate NAFLD [6]. In light of these findings, we conducted experiments to investigate the underlying mechanisms of the imbalance of mitochondrial homeostasis in NAFLD, aiming to provide additional therapeutic options for NAFLD patients.

RNA regulates gene expression through a variety of chemical modifications, among which N6-methyladenosine (m6A) is the most prevalent one. The methyltransferase-like 3 (METTL3)/methyltransferase-like 14 (METTL14) complex can incorporate m6A onto the messenger RNA (mRNA). This modification regulates the mRNA stability, splicing, processing, and nuclear export, and promotes the initiation of the mRNA translation process [7, 8]. METTL14, a common m6A methyltransferase, interacts with METTL3 to form a stable METTL3–METTL14 complex, which catalyzes the formation of m6A [9]. An overexpression of METTL14 induces the m6A modification, resulting in steatosis and hepatic fat accumulation [10]. Moreover, the activation of m6A methylation contributes to mitochondrial redox imbalance and an increase in reactive oxygen species (ROS) production [11]. Therefore, m6A methylation plays a significant role in regulating mitochondrial homeostasis. Notably, several studies have reported elevated levels of METTL14 and m6A modification in NAFLD patients [12, 13]. These findings suggest a connection between m6A modification and NAFLD, as well as mitochondrial homeostasis imbalance. However, the regulatory mechanism through which m6A modification affects NAFLD by regulating mitochondrial homeostasis has not yet been fully elucidated.

MicroRNAs (miRNAs) are small non-coding RNAs that act as post-transcriptional regulators of protein-coding genes [14]. METTL14 catalyzes the modification of primary miRNAs (pri-miRNAs) with m6A, which promotes their recognition by DiGeorge syndrome critical region 8 (DGCR8). This interaction subsequently increases the expression of mature miRNA [15]. The dysregulation of miRNAs leads to excessive ROS production, heightened endoplasmic reticulum (ER) stress, the activation of the unfolded protein response, and thus impairment of mitochondrial function [16]. For example, overexpression of the miR-21-5p sequence causes metabolic changes and increases mitochondrial respiration in H9C2 cells [17]. Conversely, the knockdown of the mitochondrial miR-1285 sequence alleviates mitochondrial respiratory dysfunction, adenosine triphosphate (ATP) deficiency, mitochondrial membrane potential (MMP) reduction, and the accumulation of mitochondrial ROS in pig jejunal epithelial cells [18]. Notably, the downregulation of the miR-34a-5p sequence mitigates NAFLD-associated fibrosis through the inhibition of hepatic stellate cell activation [19]. Additionally, the downregulation of miR-34a-5p sequence in the liver attenuates mitophagy, alleviates mitochondrial dysfunction, and repairs oxidative liver injury [20]. In our study, we further investigated the mechanism of miR-34a-5p in NAFLD, particularly its impact on regulating mitochondrial dysfunction.

The systemic RNA interference defective-1 (SID1) transmembrane family member 2 (SIDT2) is a highly glycosylated multichannel lysosomal transmembrane protein essential for maintaining the normal morphology of lysosomes [21]. The deletion of SIDT2 can lead to lipid droplet accumulation in the liver and it can hinder the maturation of autophagy-lysosomes in hepatocytes, resulting in failed breakdown of lipids into fatty acids in lysosomes, leading to the occurrence of NAFLD [22]. Moreover, silencing SIDT2 disrupts the regular mitochondrial fission and fusion processes, leading to mitochondrial dysfunction [23]. While some miRNAs have been identified as targeting SIDT2 in the context of liver diseases and cancer [24, 25], no studies have yet reported on the miR-34a-5p-mediated regulation of SIDT2. The mechanism through which m6A modification influences NAFLD by regulating mitochondrial homeostasis imbalance has not been fully explored. In this study, we reported that METTL14 exacerbates mitochondrial homeostasis imbalance, thereby promoting NAFLD. This effect is achieved by regulating the m6A modification of miR-34a, which, in turn, regulates SIDT2. Our findings provide new insights into potential treatment strategies for NAFLD.

## Materials and methods

### Experimental animal procedures

A total of 48 male C57BL/6J mice, aged 7–8 weeks and weighing between 18–22 g, were procured from Moslaite Biotechnology Co., Ltd. (Hangzhou, China) (SYXK [Zhejiang] 2022-0032). In line with experimental animal raising standards, the mice were housed at 23 ^∘^C with 50% relative humidity and with free access to water and food for one week before the experiment. Subsequently, 12 mice were randomly selected and fed with a standard diet (LAD3001-10) to serve as the control group. The remaining mice were placed on a high-fat diet (TP 26300, Trophic Animal Feed High-tech Co., Ltd., Nantong, Jiangsu, China) for eight weeks to establish the NAFLD mouse model. The body weight variations were recorded regularly. Following the high-fat diet regimen, the mice were administered lentiviral vector injections containing either short hairpin (sh)-METTL14 or sh-negative control (NC) (GENCHEM, Shanghai, China) via the tail vein at a concentration of 1 × 10^9^ TU/mL. For control purposes, an equivalent amount of saline was injected into mice through the tail vein. Eight weeks post-injection, mice were anesthetized using sodium pentobarbital (50 mg/kg). The fasting blood samples were drawn from the mice and centrifuged for 5 min at 3000 × *g*. The levels of aspartate aminotransferase (AST), alanine aminotransferase (ALT), total cholesterol (TC), and triglycerides (TG) were subsequently measured. Finally, euthanasia was carried out using an intraperitoneal injection of 150 mg/kg sodium pentobarbital [26] and the mice’s livers were excised and weighed. After weighing, the liver tissues of six randomly selected mice in each group were used for quantitative real-time polymerase chain reaction (qRT-PCR) and additional tests, while the liver tissues of the other six mice underwent hematoxylin–eosin (H&E) and other staining procedures.

### Hematoxylin–eosin, oil red O, and Masson staining assay

Liver specimens were harvested, weighed, and promptly fixed in 4% paraformaldehyde. Following paraffin embedding, the liver tissues were sectioned to a thickness of 4 µm and deparaffinized. Oil red O staining was used to visualize the lipid deposition in the liver tissues, while Masson staining was employed to assess liver fibrosis.

**Table 1:** qRT-PCR sequences of utilized primers

	**Forward primer (5**′**-3**′**)**	**Reverse primer (5**′**-3**′**)**
mmu-METTL14	GGGAAAGAAACCGATCCAATTT	AGTAAAGCCGCCTCTGTG
mmu-SIDT2	TAGTGCCTGTTACCACGTCTGC	GGATGCAGTCTGTGTAGAGCACA
mmu-pri-miR-34a	CTGGGGAGAGGCAGGACA	CGAATTCTAGAGCTCGAGGCAGG
mmu-miR-34a-5p	GCCGAGTGGCAGTGTCTTAG	CTCAACTGGTGTCGTGGA
mmu-U6	CGCACTTTACGGCTACCTCT	GCGACAAGGGAAGGGAACAA
mmu-GAPDH	AGGTCGGTGTGAACGGATTTG	GGGGTCGTTGATGGCAACA
hsa-METTL14	GGTTCTGGGGAGGGGTTG	ATGAGGCAGTGTTCCTTTGTTC
hsa-SIDT2	CCGCCGCCACCATGCGCGGCTGCCTGCG	TCAGAAGACAGGGATCTGG
hsa-pri-miR-34a	TTTAAGCTTATGCGCCCTGCC	TTTCTCGAGAGAGCTTCCGAAGTCCTGG
hsa-miR-34a-5p	GCAGTGGCAGTGTCTTAG	GGTCCAGTTTTTTTTTTTTTTTACAAC
hsa-U6	CTCGCTTCGGCAGCACA	AACGCTTCACGAATTTGCGT
hsa-GAPDH	GGAGCGAGATCCCTCCAAAAT	GGCTGTTGTCATACTTCTCATGG

qRT-PCR: Quantitative real-time polymerase chain reaction; mmu: Mus musculus; METTL14: Methyltransferase-like 14; SITD2: SID1 transmembrane family member 2; pri: Primary; miR: MicroRNA; GAPDH: Glyceraldehyde-3-phosphate dehydrogenase; hsa: *Homo sapiens*.

### Enzyme-linked immunosorbent assay

Liver tissues or serum samples were harvested from the mice. The levels of serum liver functional indices (AST and ALT) and blood lipid indices (TC and TG), as well as the expression levels of tumor necrosis factor-alpha (TNF-α), interleukin (IL)-6, and IL-1β in the liver tissues were assessed. All measurements were conducted as per the steps instructed by the respective enzyme-linked immunosorbent assay (ELISA) kits. The ELISA kits for AST (ab263882), ALT (ab282882), TC (ab285242), TNF-α (ab208348), IL-6 (ab222503), and IL-1β (ab197742) were sourced from Abcam (Cambridge, MA, USA). The ELISA kit for TG was procured from mlbio (ml095894, Shanghai, China). The optical density of each well was recorded using a microplate reader set at a wavelength of 450 nm.

### Cell culture and transfection

Human normal liver cells (L-02) were acquired from EDITGENE (EDLUCQ0106, Guangzhou, China). The cells were maintained in Roswell Park Memorial Institute 1640 medium supplemented with 10% fetal bovine serum, 100 U/mL penicillin, and 100 µg/L streptomycin. The cells were cultured in an incubator set at 37 ^∘^C with 5% CO_2_. To establish the NAFLD cell model, cells were treated with 1 mM fatty acid (comprising palmitic acid and oleic acid in a 1:2 concentration ratio) for 24 h. For the control group, an equivalent volume of culture medium was added. Plasmids, namely, small interfering (si)-METTL14-1, si-METTL14-2, si-SIDT2-1, si-SIDT2-2, and si-NC, as well as miR-34a-5p-mimic and mimic-NC, were sourced from GenePharma (Shanghai, China). These plasmids or mimics were transfected into L-02 cells using the lipofectamine 2000 transfection reagent (Invitrogen, Carlsbad, CA, USA) and cultured for 48 h before the subsequent assays.

### Quantitative real-time polymerase chain reaction

Total RNA was extracted from either mouse liver tissues or L-02 cells using the TRIzol reagent (Invitrogen). This RNA was subsequently reverse-transcribed into the complementary DNA (cDNA) utilizing a reverse transcription kit (Applied Biosystems, Carlsbad, CA, USA). For qRT-PCR, 1 µL of the cDNA was employed as per the instructions of the SYBR Green PCR Master Mix Kit (Applied Biosystems). Glyceraldehyde-3-phosphate dehydrogenase (GAPDH) and U6 served as internal reference genes [27]. The relative expression levels of METTL14, SIDT2, pri-miR-34a, and miR-34a-5p were determined using the 2^−ΔΔCt^ method [28]. The sequences of the primers utilized are presented in Table 1.

### Western blot assay

Total protein from mouse liver tissues and L-02 cells was extracted using the radioimmunoprecipitation assay lysis solution (Beyotime, Shanghai, China). Protein concentration was adjusted and quantified using the bicinchoninic acid assay kit. For protein separation, the sodium dodecyl sulfate-polyacrylamide gel electrophoresis was employed. Subsequently, the separated proteins were transferred onto polyvinylidene fluoride membranes. These membranes were then blocked with skimmed milk for 1 h, followed by overnight incubation with primary antibodies against METTL14 (1:1000; ab300104; Abcam), Fis1 (1:500; AB_1950286; GeneTex, Irvine, CA, USA), dynamin-related protein 1 (Drp1) (1:1000; ab184247; Abcam), mitofusin 2 (Mfn2) (ab50838; Abcam), SIDT2 (ab67299; Abcam), and GAPDH (1:1000; ab226408; Abcam). A 1-h incubation with the secondary antibody (1:2000; ab205718; Abcam) preceded visualization through enhanced chemiluminescence. Gray value analysis was performed using Image Lab software (Bio-Rad, Hercules, CA, USA).

### 5,5,6,6’-Tetrachloro-1,1’,3,3’-tetraethylbenzimi-dazoylcarbocyanine iodide (JC-1) staining assay

MMP was assessed using the JC-1 kit (Beyotime). Mouse liver tissues were minced, homogenized, and subjected to centrifugation at 1000 × *g* for 10 min at 4 ^∘^C. The resultant supernatant was obtained and further centrifuged for 15 min. Following this, the supernatant was discarded, and the precipitate was diluted with 1 mL of buffer to produce a single-cell suspension for subsequent assays. Both this suspension and the L-02 cells were incubated in the dark at 37 ^∘^C in an atmosphere of 5% CO_2_ and subsequently stained with JC-1 solution for 30 min. The cells were observed and photographed under a fluorescence microscope. The fluorescence intensity of JC-1 was quantitatively analyzed with the Image Pro Plus software.

### Quantification of m6A RNA methylation

Total RNA was extracted from mouse liver tissues and L-02 cells using the TRIzol reagent. The quality of the extracted RNA was assessed using NanoDrop (Thermo Fisher, Waltham, MA, USA) and further verified by 1% agarose gel electrophoresis. The levels of m6A in liver tissues and L-02 cells were quantified using the EpiQuik m6A RNA methylation quantification kit (Colorimetric, P-9005-48, Epigentek, Farmingdale, NY, USA). Absorbance was measured at a wavelength of 450 nm, and the relative m6A quantity was determined based on the standard curve.

### RNA immunoprecipitation assay

The RNA immunoprecipitation (RIP) assay was performed using the Magna RIP kit (Millipore, Billerica, MA, USA). Both tissues and cells were lysed with RIP lysis buffer (Millipore) at 4 ^∘^C using destructive ultrasonication. Endogenous DGCR8 was immunoprecipitated with the DGCR8 antibody (1:60; ab191875; Abcam) during an overnight incubation at 4 ^∘^C. After the extraction of RNAs, the levels of pri-miR-34a were analyzed by qRT-PCR.

For the m6A RNA binding assay, RNAs were extracted from tissues and cells and treated with DNase I, followed by sonication to fragment the RNAs. The m6A antibody (ab264408; Abcam) was bound to magnetic beads, and RNA fragments were subsequently immunoprecipitated using the RIP assay. After a 1.5-h treatment of the magnetic beads with proteinase K, RNAs were extracted, and the enrichment of pri-miR-34a was analyzed using qRT-PCR.

### Bioinformatics

The microRNA Target Prediction Database (miRDB) (http://mirdb.org/) [29], TargetScan7.2 (http://www.targetscan.org/vert_72/) [30], and miRWalk (http://mirwalk.umm.uni-heidelberg.de/) [31] were used to predict the downstream target genes of miR-34a-5p. The TargetScan7.2 database was used to predict the binding site between miR-34a-5p and SIDT2.

### Dual-luciferase reporter gene assay

The SIDT2 wild type (WT) was constructed by amplifying the SIDT2 3’-UTR fragment that contains the miR-34a-5p binding site. The SIDT2 mutant type (MUT) was constructed by site-directed mutagenesis. Both the SIDT2-WT and SIDT2-MUT sequences were then cloned into the pGL vector. These constructs were co-transfected into L-02 cells along with either the miR-34a-5p-mimic or mimic-NC using the lipofectamine 2000 transfection reagent. After 48 h of culture, the relative activity of luciferase was determined using the dual-luciferase kit (Beyotime).

### Ethical statement

The animal experiment received approval from the Animal Ethics Committee of Hunan Aerospace Hospital (Approval number: HNHTYY20221108LLSH-025-01). All experimental procedures involving animals were performed in accordance with the standards set forth in the Guidelines for the Care and Use of Laboratory Animals [32].

**Figure 1.**
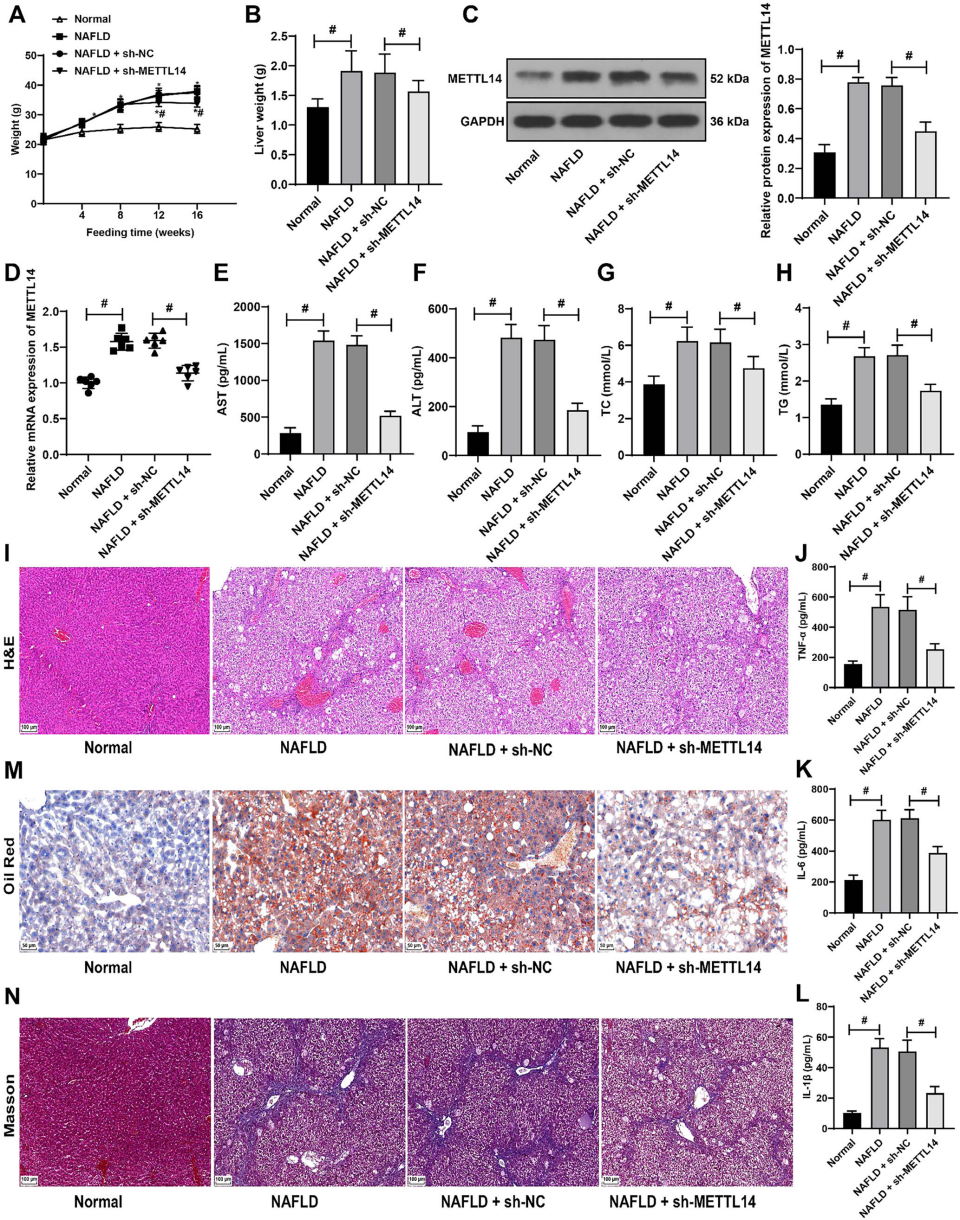
**Elevated METTL14 expression in NAFLD mice and its attenuation through silencing.** The NAFLD mouse model was established by high-fat diet. After eight weeks of high-fat diet feeding, the expression of METTL14 was silenced by a tail vein injection of lentivirus packaged with sh-METTL14 (with sh-NC as control). (A) Regular recording of the mice’s body weight throughout the feeding period; (B) Liver weight recorded at the conclusion of feeding regimen; (C) Western blot assay showing METTL14 protein expression in liver tissues; (D) qRT-PCR analysis of *METTL14* mRNA levels in liver tissues; (E–H) ELISA measurements of serum levels of AST (E), ALT (F), TC (G), and TG (H); (I) H&E staining assay revealing the pathological alterations in mouse liver tissues; (J–L) ELISA measurements of TNF-α (J), IL-6 (K), and IL-1β (L) levels in mouse liver tissues; (M) Lipid accumulation in the liver visualized using Oil Red O staining; (N) Liver fibrosis assessed by Masson staining. For panels A and B and E-H the number of mice was *n* ═ 12; for panels C and D and I–N the number of mice was *n* ═ 6. Data are presented as mean ± SD. Data in panel A were analyzed using the two-way ANOVA test, while data in panels B–L were evaluated with one-way ANOVA, followed by Tukey’s post-hoc test. In A: * indicates comparison with the control group, # indicates comparison with the sh-NC group, both at *P* < 0.05. In B–L: # signifies a *P* < 0.05. METTL14: Methyltransferase-like 14; NAFLD: Non-alcoholic fatty liver disease; sh: Short hairpin RNA; NC: Negative control; qRT-PCR: Quantitative real-time polymerase chain reaction; ELISA: Enzyme-linked immunosorbent assay; AST: Aspartate aminotransferase; ALT: Alanine aminotransferase; TC: Total cholesterol; TG: Triglycerides; H&E: Hematoxylin–eosin; TNF-α: Tumor necrosis factor-alpha; IL: Interleukin; ANOVA: Analysis of variance; GAPDH: Glyceraldehyde-3-phosphate dehydrogenase; mRNA: Messenger RNA; SD: Standard deviation.

### Statistical analysis

Data analyses were conducted using the GraphPad Prism 8.0 software (GraphPad Software Inc., San Diego, CA, USA). All measurement data are presented as mean ± standard deviation (SD). Pairwise data comparisons were assessed using the *t*-test, while multigroup data comparisons were evaluated using one-way or two-way analysis of variance (ANOVA), followed by post-hoc Tukey’s multiple comparison test. A *P* value of < 0.05 was considered statistically significant.

**Figure 2.**
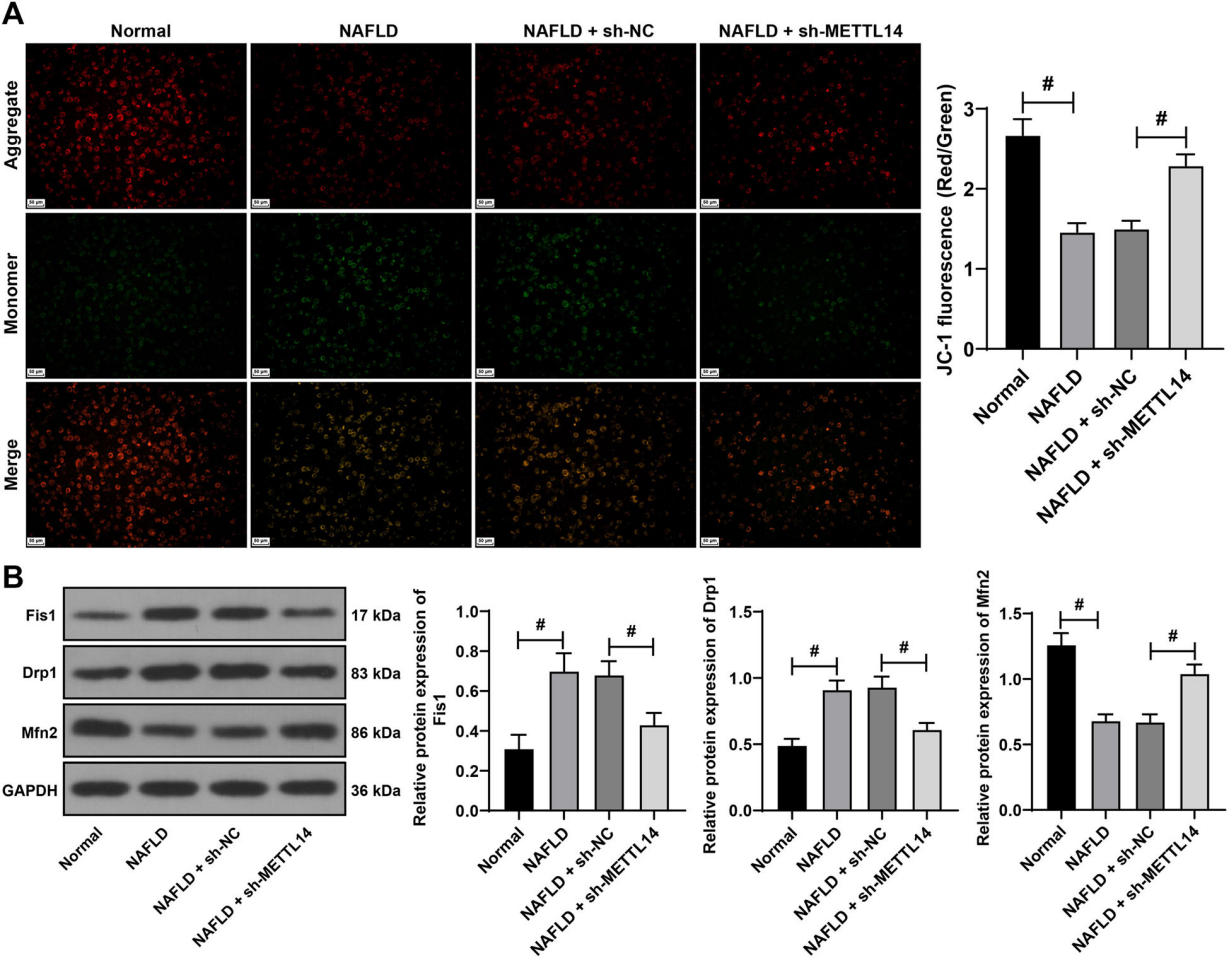
**METTL14 silencing alleviates mitochondrial homeostasis imbalance in NAFLD mice.** The NAFLD mouse model was established by high-fat diet. After eight weeks of high-fat diet feeding, the expression of METTL14 was silenced by a tail vein injection of lentivirus packaged with sh-METTL14 (with sh-NC as control). (A) The JC-1 staining assay employed to assess mitochondrial membrane potential in mouse liver tissues; (B) Western blotting assay used to detect the protein expression levels of Fis1, Drp1, and Mfn2 in liver tissues. For each panel the number of mice was *n* ═ 6. Data are presented as mean ± SD. Data were analyzed using the one-way ANOVA test, followed by Tukey’s post-hoc test. ^#^*P* < 0.05. METTL14: Methyltransferase-like 14; NAFLD: Non-alcoholic fatty liver disease; sh: Short hairpin RNA; NC: Negative control; JC-1: 5,5,6,6’-tetrachloro-1,1’,3,3’-tetraethylbenzimi-dazoylcarbocyanine iodide staining assay; Fis1: Fission 1 protein; Drp1: Dynamin-related protein 1; Mfn2: Mitofusin 2; ANOVA: Analysis of variance; GAPDH: Glyceraldehyde-3-phosphate dehydrogenase; SD: Standard deviation.

**Figure 3.**
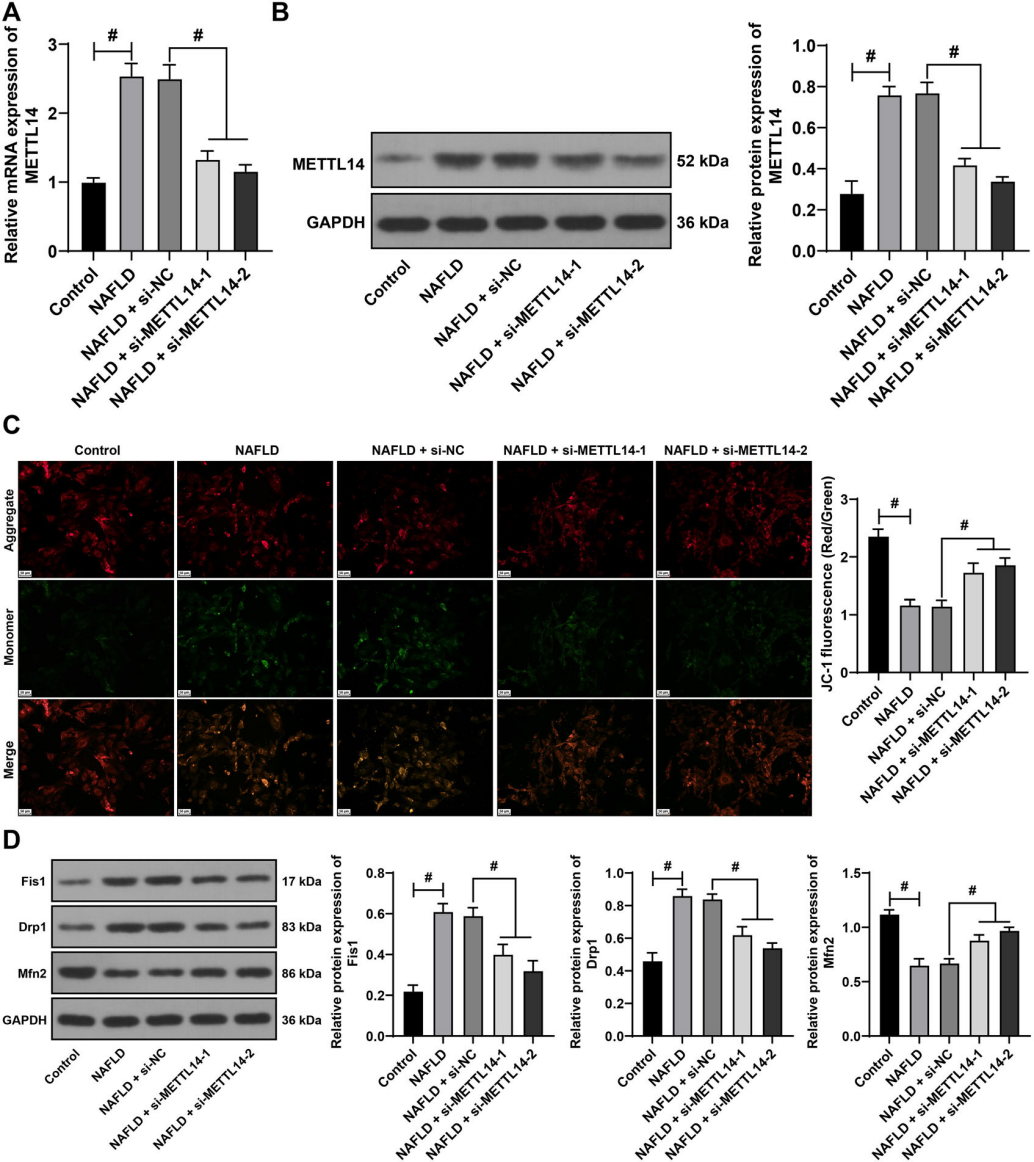
**METTL14 silencing alleviates mitochondrial homeostasis imbalance in L-02 cells.** L-02 cells were treated with fatty acids to establish the NAFLD cell model, and si-METTL14-1 and si-METTL14-2 (si-NC as control group) were transfected into the L-02 cells to reduce the expression of METTL14. (A and B) qRT-PCR (A) and western blot assay (B) used to assess METTL14 expression in the cells; (C) JC-1 staining assay employed to evaluate mitochondrial membrane potential; (D) Western blotting assay used to detect the protein levels of Fis1, Drp1, and Mfn2 in the cells. All cell experiments were performed three times independently. Data are presented as mean ± SD. Data were analyzed using the one-way ANOVA test, followed by Tukey’s post-hoc test. ^#^*P* < 0.05. METTL14: Methyltransferase-like 14; L-02 cells: Human normal liver cells; NAFLD: Non-alcoholic fatty liver disease; si: Small interfering; NC: Negative control; qRT-PCR: Quantitative real-time polymerase chain reaction; JC-1: 5,5,6,6’-Tetrachloro-1,1’,3,3’-tetraethylbenzimi-dazoylcarbocyanine iodide staining assay; Fis1: Fission 1 protein; Drp1: Dynamin-related protein 1; Mfn2: Mitofusin 2; ANOVA: Analysis of variance; mRNA: Messenger RNA; GAPDH: Glyceraldehyde-3-phosphate dehydrogenase; SD: Standard deviation.

**Figure 4.**
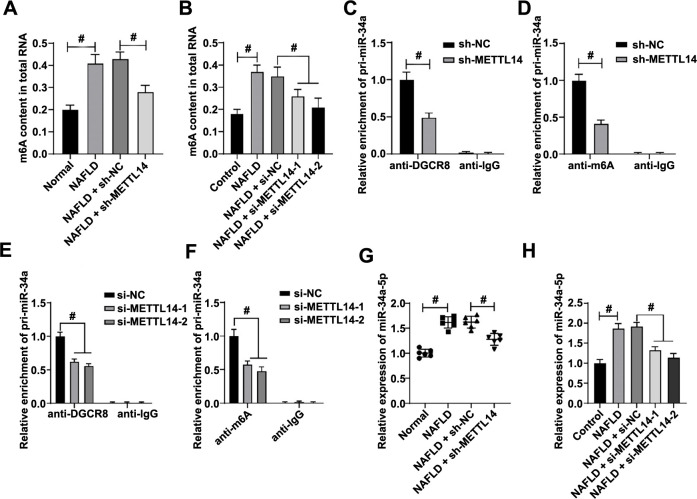
**METTL14-mediated m6A modification promotes DGCR8 binding to pri-miR-34a and increases the expression of mature miR-34a-5p.** Samples of liver tissues from NAFLD mice and L-02 cells were collected. (A and B) RNA m6A quantification employed to determine m6A levels in mouse liver tissues (A) and L-02 cells (B); (C) qRT-PCR utilized to measure the levels of pri-miR-34a bound to DGCR8 in mouse liver tissues; (D) qRT-PCR utilized to measure the levels of m6A-modified pri-miR-34a in liver tissues; (E) qRT-PCR utilized to measure the level of pri-miR-34a bound to DGCR8 in L-02 cells; (F) qRT-PCR utilized to measure the levels of m6A-modified pri-miR-34a in L-02 cells; (G and H) qRT-PCR utilized to measure the expression of miR-34a-5p in mouse liver tissues (G) and in L-02 cells (H). For panels A, C, D, and G, the number of mice was *n* ═ 6. Experiments for panels B, E, F, and H were performed three times independently. Data are presented as mean ± SD. Data in panels A, B, G, and H were analyzed using the one-way ANOVA test. Data in panels C–F were analyzed using the two-way ANOVA test, followed by Tukey’s post-hoc test. ^#^*P* < 0.05. METTL14: Methyltransferase-like 14; m6A: N6-methyladenosine; DGCR8: DiGeorge syndrome critical region 8; pri: Primary; miR: MicroRNA; NAFLD: Non-alcoholic fatty liver disease; L-02: Human normal liver cells; qRT-PCR: Quantitative real-time polymerase chain reaction; ANOVA: Analysis of variance; sh: Short hairpin; NC: Negative control; IgG: Immunoglobulin G; SD: Standard deviation.

## Results

### METTL14 is highly expressed in NAFLD mice and METTL14 silencing alleviates liver injury in NAFLD mice

The mouse model of NAFLD was established using a high-fat diet to investigate the significance of METTL14 in NAFLD. After eight weeks on this diet, METTL14 expression was inhibited through a tail vein injection of lentivirus-packaged sh-METTL14. The body weight of mice was recorded regularly during the feeding process. It was found that the body weight of NAFLD mice began to increase significantly after the 4th week of high-fat diet feeding, with a significant increase in the weight of the liver also being observed (*P* < 0.05, Figure 1A and 1B). Concurrently, METTL14 was abundantly expressed in the NAFLD mice (*P* < 0.05, Figure 1C and 1D), accompanied by significantly elevated serum levels of AST, ALT, TC, and TG (*P* < 0.05, Figure 1E–1H). Histological examination using the H&E staining revealed significant pathological alterations in the liver tissues (Figure 1I), along with significant increases in the TNF-α, IL-6, and IL-1β levels (*P* < 0.05, Figure 1J–1L). A significant increase in the number of lipid droplets accumulated in the NAFLD liver was noted (Figure 1M), and Masson staining highlighted fibrotic changes in the liver of NAFLD mice (Figure 1N). Upon METTL14 silencing (*P* < 0.05, Figure 1C and 1D), the body weight of the mice was significantly reduced starting from the 12th week (*P* < 0.05, Figure 1A and 1B). Compared to the NAFLD mice group, METTL14-silenced mice exhibited decreased levels of AST, ALT, TC, and TG (*P* < 0.05, Figure 1E–1H). Their liver pathological changes showed significant improvements (Figure 1I), with reduced levels of TNF-α, IL-6, and IL-1β (*P* < 0.05, Figure 1J–1L). Furthermore, reductions in the number of lipid droplets in the liver were observed (Figure 1M), and the degree of liver fibrosis was mitigated (Figure 1N). These results suggested that silencing METTL14 effectively slowed down the body weight gain, and alleviated liver injury, inflammation, steatosis, and tissue structural damage induced by a high-fat diet.

### METTL14 silencing alleviates the imbalance of mitochondrial homeostasis in NAFLD mice

Mitochondrial homeostasis imbalance is the key cause of NAFLD [33, 34]. Previous studies have shown that METTL14-mediated m6A modification can promote mitochondrial fission [35]. Based on these findings, we speculated that METTL14 may improve liver injury in NAFLD mice by regulating mitochondrial homeostasis. The detection results of NAFLD mice revealed a decrease in MMP (*P* < 0.05, Figure 2A), an increase in the expression levels of mitochondrial fission proteins, namely, mitochondrial fission 1 protein (Fis1) and Drp1, and a decrease in the fusion protein Mfn2 expression (*P* < 0.05, Figure 2B). After METTL14 silencing, there was an increase in MMP (*P* < 0.05, Figure 2A), a decrease in Fis1 and Drp1 expression levels, and an increase in Mfn2 expression (*P* < 0.05, Figure 2B). These results verified our speculation at the preliminary level.

### METTL14 silencing alleviates the imbalance of mitochondrial homeostasis in L-02 cells

We next strived to verify that silencing METTL14 can alleviate mitochondrial homeostasis imbalance in NAFLD through in vitro assays. Fatty acid-treated L-02 cells were used to establish a cell model of NAFLD. After the fatty acid treatment, the results revealed an increase in METTL14 (*P* < 0.05, Figure 3A and 3B), a decrease of MMP (*P* < 0.05, Figure 3C), an increase in the levels of mitochondrial fission proteins Fis1 and Drp1, and a decrease in the fusion protein Mfn2 levels (*P* < 0.05, Figure 3D). To suppress the METTL14 expression, si-METTL14 was transfected into L-02 cells, which led to significant inhibition (*P* < 0.05, Figure 3A and 3B). Following this treatment, an increase of MMP was observed (*P* < 0.05, Figure 3C), Fis1 and Drp1 levels decreased, and Mfn2 levels increased (*P* < 0.05, Figure 3D). These results indicated that the mitochondrial homeostasis imbalance occurred in the NAFLD cell model and that the inhibition of METTL14 expression improved the mitochondrial homeostasis.

### METTL14-mediated m6A modification promotes the DGCR8 binding to pri-miR-34a and increases the expression of mature miR-34a-5p

Further investigation was conducted into the specific mechanism through which METTL14 affects mitochondrial homeostasis. As a methyltransferase, METTL14 catalyzes m6A modification to promote miRNA maturation [36]. We assessed the expression of m6A in liver tissues of NAFLD mice and in cell models. The detection results showed that m6A levels were highly elevated. When METTL14 was inhibited, m6A levels decreased significantly (*P* < 0.05, Figure 4A and 4B), suggesting the role of METTL14 in modulating m6A methylation in NAFLD. It has been reported that METTL14 can promote the m6A modification of pri-miR-34a, thereby promoting the expression of mature miR-34a-5p [37]. Given that the miR-34a-5p is highly expressed in NAFLD [19], we speculated that METTL14 might regulate the expression of miR-34a-5p in both the NAFLD animal and cell models. Subsequent to METTL14 expression inhibition, the level of pri-miR-34a bound to DGCR8 was significantly decreased, coupled with a significant decrease in the level of m6A-modified pri-miR-34a (*P* < 0.05, Figure 4C–4F). Moreover, upon evaluating miR-34a-5p expression in mouse liver tissues and L-02 cells, we found that its levels were heightened in NAFLD. Inhibiting METTL14 expression led to a significant decrease in miR-34a-5p levels (*P* < 0.05, Figure 4G and 4H). These results suggested that METTL14-mediated m6A modification could promote the binding of DGCR8 to pri-miR-34a, thereby increasing mature miR-34a-5p expression.

### The overexpression of miR-34a-5p partially reverses the alleviative effect of METTL14 silencing on mitochondrial homeostasis imbalance

**Figure 5.**
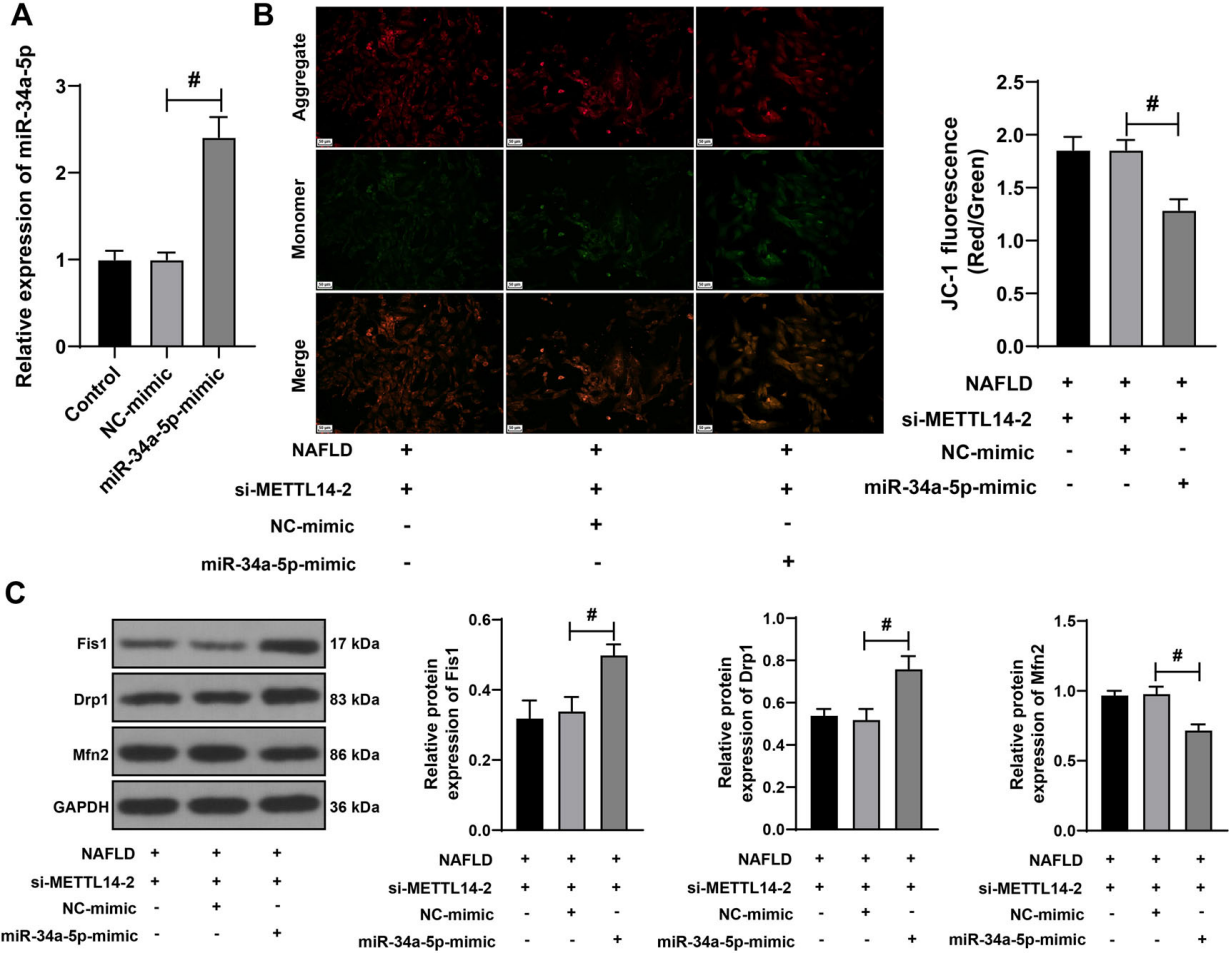
**Overexpression of miR-34a-5p partially reverses the alleviative effect of METTL14 silencing on mitochondrial homeostasis imbalance.** L-02 cells underwent transfection with miR-34a-5p-mimic (with NC-mimic as control). (A) qRT-PCR utilized to verify the transfection efficiency of miR-34a-5p-mimic. The combined experiment was conducted with si-METTL14-2, which displayed better silencing efficiency; (B) JC-1 staining assay utilized to assess the mitochondrial membrane potential; (C) Western blotting assay utilized to determine the protein levels of Fis1, Drp1, and Mfn2 in the cells. Cell experiments were performed three times independently. Data are presented as mean ± SD. Data in panels A–F were analyzed using the one-way ANOVA test, followed by Tukey’s post-hoc test. ^#^*P* < 0.05. miR: MicroRNA; METTL14: Methyltransferase-like 14; L-02: Human normal liver cells; NC: Negative control; qRT-PCR: Quantitative real-time polymerase chain reaction; si: Small interfering; JC-1: 5,5,6,6’-Tetrachloro-1,1’,3,3’-tetraethylbenzimi-dazoylcarbocyanine iodide staining assay; Fis1: Fission 1 protein; Drp1: Dynamin-related protein 1; Mfn2: Mitofusin 2; ANOVA: Analysis of variance; NAFLD: Non-alcoholic fatty liver disease; GAPDH: Glyceraldehyde-3-phosphate dehydrogenase; SD: Standard deviation.

We further investigated whether METTL14 regulates mitochondrial homeostasis through the regulation of miR-34a-5p expression. L-02 cells were transfected with miR-34a-5p-mimic to induce the overexpression of miR-34a-5p (*P* < 0.05, Figure 5A). This was done in combination with si-METTL14-2, which exhibited superior transfection efficiency. Following the overexpression of miR-34a-5p, the MMP decreased (*P* < 0.05, Figure 5B). The protein levels of Fis1 and Drp1 increased, while the protein expression of Mfn2 significantly decreased (*P* < 0.05, Figure 5C). These results suggested that the overexpression of miR-34a-5p could partially reverse the alleviative effect of METTL14 silencing on the mitochondrial homeostasis imbalance.

**Figure 6.**
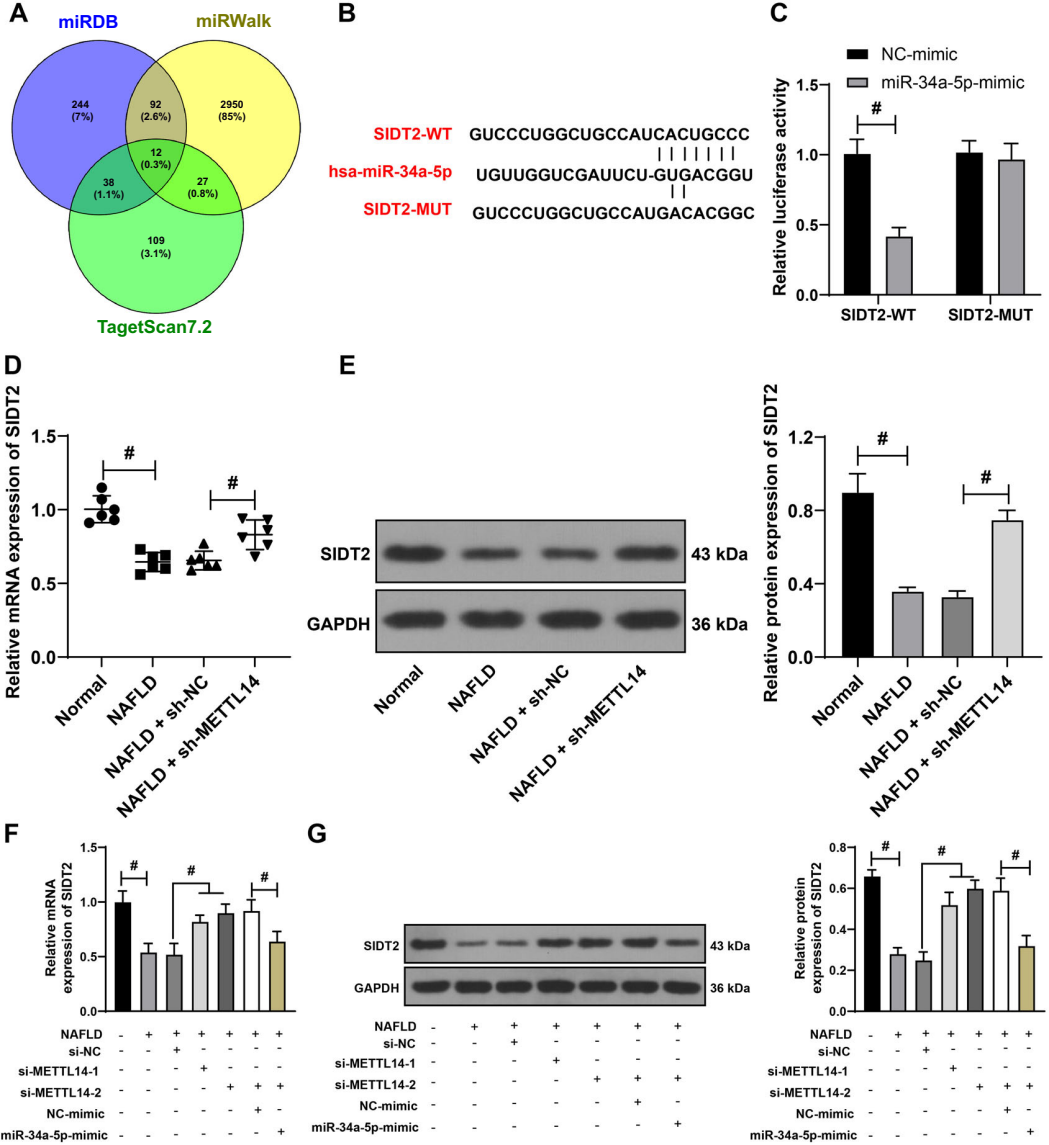
**Identification of SIDT2 as a downstream target gene of miR-34a-5p.** (A) Online databases TargetScan7.2, miRWalk, and miRDB employed to predict potential downstream targets of miR-34a-5p; (B) TargetScan7.2 utilized to predict the binding sequence between miR-34a-5p and SIDT2; (C) A dual-luciferase reporter gene assay conducted to verify the relationship between miR-34a-5p and SIDT2. The liver tissues of NAFLD mice and L-02 cells were harvested; (D) qRT-PCR utilized to measure the mRNA expression of *SIDT2* in mouse liver tissues; (E) Western blot assay utilized to determine the expression of SIDT2 protein in mouse liver tissues; (F) qRT-PCR utilized to measure the mRNA expression of *SIDT2* in L-02 cells; (G) Western blot assay utilized to determine the expression of SIDT2 protein in L-02 cells. For panels C, F, and G, the cell experiments were performed three times independently. For panels D and E, the number of mice was *n* ═ 6. Data in panel C were analyzed using the two-way ANOVA test. Data in panels D–G were analyzed using the one-way ANOVA test, followed by Tukey’s post-hoc test. ^#^*P* < 0.05. SIDT2: SID1 transmembrane family member 2; miR: MicroRNA; miRDB: MicroRNA target prediction database; NAFLD: Non-alcoholic fatty liver disease; L-02: Human normal liver cells; qRT-PCR: Quantitative real-time polymerase chain reaction; mRNA: Messenger RNA; ANOVA: Analysis of variance; WT: Wild-type; hsa: *Homo sapiens*; MUT: Mutant type; NC: Negative control; sh: Short hairpin; METTL14: Methyltransferase-like 14; GAPDH: Glyceraldehyde-3-phosphate dehydrogenase; si: Small interfering.

### SIDT2 is identified as a downstream target gene of miR-34a-5p

Next, to elucidate the downstream regulatory mechanism of miR-34a-5p, we employed online databases including TargetScan7.2, miRWalk, and miRDB to screen for potential target genes. Among the identified candidates, SIDT2 emerged as a common target (Figure 6A). Through literature review, we found that SIDT2 plays a role in lipid metabolism, and its deletion significantly affects NAFLD progression [22, 38]. TargetScan7.2 prediction revealed that miR-34a-5p had a binding sequence with SIDT2 (Figure 6B), which was subsequently confirmed through the dual-luciferase reporter gene experiment (*P* < 0.05, Figure 6C). SIDT2 was poorly expressed in liver tissues of NAFLD mice and fatty acid-treated L-02 cells. However, its expression was significantly increased after METTL14 silencing (*P* < 0.05, Figure 6D–6G). Moreover, cellular assays indicated that, after METTL14 silencing, the overexpression of miR-34a-5p led to a significant decrease in SIDT2 expression (*P* < 0.05, Figure 6F and 6G). These results suggested that SIDT2 acts as a downstream target gene of miR-34a-5p. Furthermore, within the context of NAFLD, miR-34a-5p appears capable of targeting and inhibiting SIDT2 expression.

**Figure 7.**
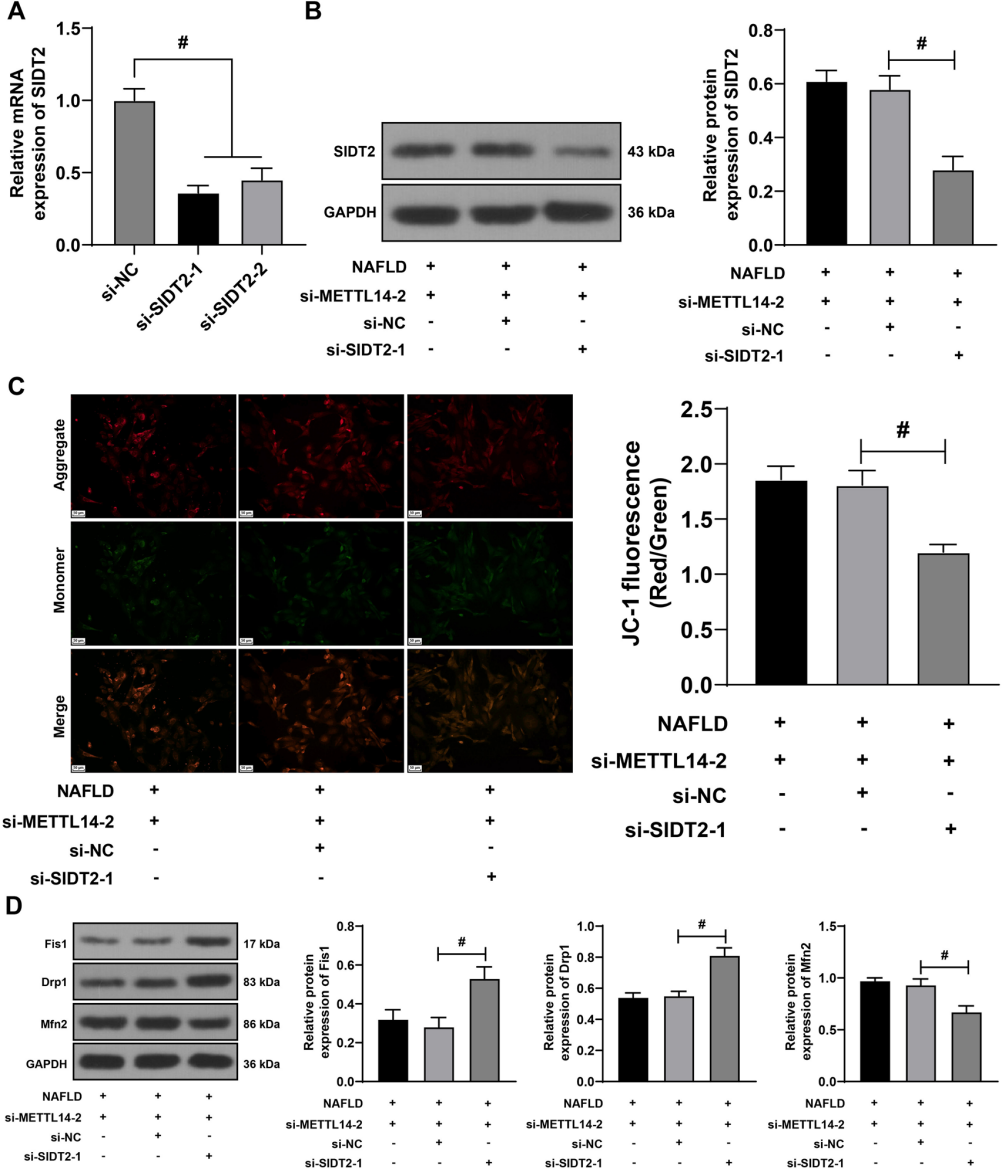
**Inhibition of SIDT2 expression partially reverses the alleviative effect of METTL14 silencing on mitochondrial homeostasis imbalance.** L-02 cells were transfected with si-SIDT2-1 and si-SIDT2-2 (with si-NC as control). (A) qRT-PCR utilized to verify the transfection efficiency. For combined experiments, si-METTL14-2 was paired with si-SIDT2-1 due to its higher transfection efficiency; (B) Western blot assay utilized to determine the protein levels of SIDT2 in the L-02 cells; (C) JC-1 staining assay employed to evaluate mitochondrial membrane potential; (D) Western blot assay utilized to determine the protein levels of Fis1, Drp1, and Mfn2 in the L-02 cells. All cell experiments were performed three times independently. Data are presented as mean ± SD. Data in panels A–D were analyzed using the one-way ANOVA test, followed by Tukey’s post-hoc test. ^#^*P* < 0.05. SIDT2: SID1 transmembrane family member 2; METTL14: Methyltransferase-like 14; L-02: Human normal liver cells; si: Small interfering; NC: Negative control; qRT-PCR: Quantitative real-time polymerase chain reaction; JC-1: 5,5,6,6’-tetrachloro-1,1’,3,3’-tetraethylbenzimi-dazoylcarbocyanine iodide staining assay; Fis1: Fission 1 protein; Drp1: Dynamin-related protein 1; Mfn2: Mitofusin 2; ANOVA: Analysis of variance; mRNA: Messenger RNA; GAPDH: Glyceraldehyde-3-phosphate dehydrogenase; NAFLD: Non-alcoholic fatty liver disease; SD: Standard deviation.

### Inhibition of SIDT2 partially reverses the alleviative effect of METTL14 silencing on mitochondrial homeostasis imbalance

Subsequently, we further sought to verify whether SIDT2 was involved in METTL14’s regulation of mitochondrial homeostasis. L-02 cells were transfected with si-SIDT2-1 and si-SIDT2-2 to suppress the SIDT2 expression (*P* < 0.05, Figure 7A and 7B). For combination experiments, we utilized si-METTL14-2 and si-SIDT2-1, both of which exhibited higher transfection efficiencies. Following the SIDT2 suppression, the MMP decreased (*P* < 0.05, Figure 7B). The protein levels of Fis1 and Drp1 increased, while the protein expression of Mfn2 significantly decreased (*P* < 0.05, Figure 7C). These results suggested that the inhibition of SIDT2 could partially reverse the alleviative effect of METTL14 silencing on the mitochondrial homeostasis imbalance.

## Discussion

**Figure 8.**
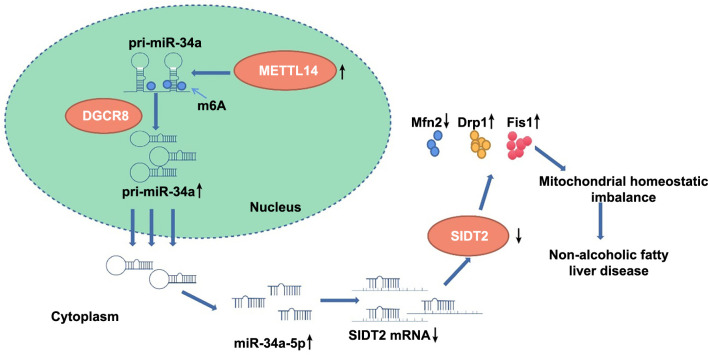
**METTL14’s role in non-alcoholic fatty liver disease through mitochondrial homeostasis regulation.** METTL14 upregulation catalyzes the m6A modification of pri-miR-34a, enhancing the binding of pri-miR-34a to DGCR8, which in turn increases the expression of mature miR-34a-5p. This miR-34a-5p then binds to SIDT2 mRNA, thereby suppressing SIDT2 expression. As a result, the expression of mitochondrial fission proteins Fis1 and Drp1 is upregulated, while the expression of the mitochondrial fusion protein Mfn2 is downregulated. This cascade contributes to an imbalance in mitochondrial homeostasis, aggravating NAFLD. METTL14: Methyltransferase-like 14; m6A: N6-methyladenosine; pri: Primary; miR: MicroRNA; DGCR8; DiGeorge syndrome critical region 8; SIDT2: SID1 transmembrane family member 2; mRNA: Messenger RNA; Fis1: Fission 1 protein; Drp1: Dynamin-related protein 1; Mfn2: Mitofusin 2; NAFLD: Non-alcoholic fatty liver disease; pre: Precursor.

NAFLD is characterized by excessive accumulation of TG, inflammation, injury, and apoptosis [39]. Mitochondrial dysfunction is one of the most significant features of NAFLD, and significant alterations of mitochondrial parameters have been observed in NAFLD patients [40]. While prior studies have largely focused on nuclear receptors, compounds, and enzymes related to mitochondrial metabolism as potential targets for mitochondrial dysfunction in NAFLD, the role of m6A modifying enzymes in mitochondrial function has rarely been explored [41]. In this study, we made the following observations: 1) METTL14 catalyzed the m6A modification of pri-miR-34a to promote the binding of pri-miR-34a to DGCR8, which, in turn, increased the expression of mature miR-34a-5p; 2) miR-34a-5p bound to SIDT2 mRNA, suppressing SIDT2 expression; and 3) inhibition of SIDT2 promoted the expression of mitochondrial fission proteins Fis1 and Drp1 and inhibited the expression of mitochondrial fusion protein Mfn2, leading to imbalance of mitochondrial homeostasis and aggravation of NAFLD (Figure 8).

Mitochondrial dysfunction is characterized by an increase in ROS, a reduction in MMP, elevated levels of mitochondrial fission proteins Fis1 and Drp1, decreased levels of mitochondrial fusion protein Mfn2, and mitochondrial membrane damage. Such alterations lead to hepatic lipid accumulation in NAFLD [34]. Aberrant upregulation of METTL14 and m6A resulted in the suppression of hepatic lipolysis, adipose tissue expansion, and metabolic disorders [42]. METTL14 amplifies the m6A modification of Fis1, thereby enhancing its expression and promoting mitochondrial fission [35]. Based on these observations, we hypothesized that METTL14 might influence NAFLD by mediating m6A modifications, subsequently disrupting mitochondrial homeostasis. Serum levels of AST, ALT, TC, and TG serve as common diagnostic indicators of steatosis, liver fibrosis, liver cirrhosis, and NAFLD [43]. Overexpression of METTL14 has increased the levels of AST, ALT, TG, TC, pro-inflammatory factors, and lipid accumulation, thereby promoting chronic inflammation and cellular apoptosis, typical features of NAFLD [10]. In our study, METTL14 was found to be overexpressed in NAFLD mice. Concomitantly, serum levels of AST, ALT, TC, TG, and pro-inflammatory factors TNF-α, IL-6, and IL-1β, were significantly elevated. Silencing METTL14 counteracted these increases, attenuating liver inflammation and liver tissue damage in mice. Additionally, in both NAFLD mice and in vitro cell models, MMP was observed to decrease, the expression levels of mitochondrial fission proteins Fis1 and Drp1 increased, and the expression levels of mitochondrial fusion protein Mfn2 decreased. Silencing METTL14 reversed the changes above, suggesting that the suppression of METTL14 expression could maintain mitochondrial homeostasis and reduce liver injury in NAFLD.

The methyltransferase catalyzes m6A modification and promotes the binding between DGCR8 and pri-miRNA, leading to miRNA maturation [44]. Previous studies highlighted that METTL14-mediated m6A modification improves the binding of pri-miR-375 to DGCR8. This subsequently fosters the conversion of pri-miR-375 into its mature form, miR-375, which then triggers neuronal apoptosis in the spinal cord [45]. Similarly, METTL14-mediated m6a methylation positively regulates the maturation of miR-34 and promotes the expression of miR-34a-5p [37]. Our experiments confirm these findings, indicating that METTL14 suppression reduces the binding of pri-miR-34a to DGCR8 and attenuates the expression of miR-34a-5p. This suggests that METTL14-mediated m6A modification can potentiate the binding of DGCR8 to pri-miR-34a, thereby elevating the levels of mature miR-34a-5p. Moreover, a previous study has shown that miR-34a-5p is overexpressed in NAFLD, where it induces glucose metabolism and lipogenesis in hepatocytes [46]. The overexpression of miR-34a-5p mediates mitophagy disruption, resulting in the accumulation of dysfunctional mitochondria, which subsequently leads to oxidative liver injury [20]. In conditions where miR-34a-5p was overexpressed, we observed a decline in MMP, an increase in Fis1 and Drp1 protein levels, and a significant decrease in Mfn2 protein. These observations suggest that elevating miR-34a-5p levels counterbalances the beneficial effects of METTL14 silencing on mitochondrial homeostasis.

Database analysis showed that SIDT2 may be a downstream target of miR-34a-5p. In SIDT2-deficient mice, a large accumulation of lipid droplets was found in the liver tissues, as well as elevated levels of ALT and AST, suggesting the onset of hepatocellular injury [22]. Furthermore, these SIDT2-deficient mice exhibited ER stress and unfolded protein response, exacerbating the progression of NAFLD [38]. The SIDT2 deletion impairs the fusion between autophagosomes and lysosomes. This results in a decrease in the number of acidic lysosomes and compromised lysosomal function, subsequently causing the mitochondrial homeostasis imbalance [47, 48]. The expression of SIDT2 was downregulated in NAFLD mouse liver tissues and L-02 cells, and silencing METTL14 significantly increased SIDT2. Upon overexpressing miR-34a-5p under these conditions, we observed a reduction in SIDT2 expression. This suggests that METTL14 augments miR-34a-5p expression, which in turn suppresses SIDT2 expression. After inhibiting SIDT2 expression, a decrease in MMP was noted, accompanied by elevated expression levels of Fis1 and Drp1 proteins, while the expression of Mfn2 protein decreased. These findings suggest that inhibiting SIDT2 counteracts the beneficial effects of METTL14 silencing on mitochondrial homeostasis imbalance.

Our study possesses several limitations. Firstly, while METTL14 can catalyze m6A modification to promote the maturation of multiple miRNAs, our study only explored miR-34a-5p, omitting the potential effects of other miRNAs on mitochondrial homeostasis and NAFLD. Secondly, except for SIDT2, we did not explore other downstream target genes of miR-34a-5p. Thirdly, other m6A-modifying enzymes were not studied. Lastly, our focus was limited to the m6A modification of miR-34a mediated by METTL14. We did not investigate possible m6A modifications on the mRNA of SIDT2, its role in NAFLD, or whether other genes might be regulated by m6A modification in the context of NAFLD. Moving forward, we plan to expand our research to encompass other miRNAs and target genes, delve deeper into the roles of other m6A modifying enzymes like METTL3 in mitochondrial homeostasis imbalance and NAFLD, and further elucidate the regulatory mechanism of SIDT2 in NAFLD. We also aim to explore genome-wide and transcriptome-wide epitranscriptomic profiling of miRNA and mRNA, to discern if other miRNAs or mRNAs are involved in the epitranscriptomic regulation of NAFLD.

## Conclusion

In summary, our study revealed that in NAFLD, METTL14 is upregulated and subsequently facilitates the binding of pri-miR-34a to DGCR8, leading to an increase in mature miR-34a-5p expression. This elevates miR-34a-5p then suppresses SIDT2 expression, resulting in an imbalance in mitochondrial homeostasis that aggravates NAFLD. Our study revealed a novel mechanism of METTL14 in NAFLD, offering potential targets and new directions for disease treatment.

## Data Availability

The datasets generated or analyzed during the current study are available from the corresponding author upon reasonable request.
